# Identification of selection signatures in Iranian dromedary and Bactrian camels using whole genome sequencing data

**DOI:** 10.1038/s41598-022-14376-7

**Published:** 2022-06-10

**Authors:** Reza Khalkhali-Evrigh, Nemat Hedayat, Liang Ming, Jirimutu

**Affiliations:** 1 Department of Animal Science, Faculty of Agriculture and Natural Recourses, University of Mohaghegh Ardabili, Ardabil, Iran; 2 College of Food Science and Engineering, Inner Mongolia Agricultural University, Huhhot, China

**Keywords:** Animal breeding, Genome, Genomics, Sequencing, Genomic analysis

## Abstract

The Old World camels play an important role as one of the main food sources in large parts of Asia and Africa. Natural selection combined with artificial selection by human has affected parts of the domestic animal genome for adapting them to their habitats and meeting human needs. Here, we used whole genome sequencing data of 34 camels (including 14 dromedaries and 20 Bactrian camels) to identify the genomic signature of selection in the Iranian dromedary (ID) and Bactrian camels (IB). To detect the mentioned regions, we used two methods including population differentiation index (Fst) and cross-population extended haplotype homozygosity (XP-EHH) with 50 kb sliding window and 25 kb step size. Based on gene ontology analysis on the candidate genes identified for IB camels, we found GO terms associated with lung development, nervous system development, immune system and behavior. Also, we identified several genes related to body thermoregulation (ZNF516), meat quality (ANK1 and HSPA13), and high-altitude adaptation (OPA1) for IB camels. In the list of detected candidate genes under selection in ID camels, the genes related to energy metabolism (BDH1), reproduction (DLG1, IMMP2L and FRASI), long-term memory (GRIA1), kidney (SLC12A1), lung development (EMILIN2 and FBN1) and immunity (SOCS2, JAK1, NRROS and SENP1) were found. Our findings, along with further studies in this field, will strengthen our knowledge about the effect of selection on the camelid genome under different geographical, climatic and even cultural conditions.

## Introduction

Human beings started to lead a settled life based on agriculture about 12,000 years ago (ya) in Near East^1^. The shift of the human lifestyle from hunter-gatherer to farmer changed the human attitude about the environment. One of the most important and influential events in the history of our species was the attempt to domesticate plants and animals. Following dogs as the first domesticated animal (~ 23,000 ya in Siberia)^2^ which helped increase human security, animals such as sheep (~ 11,000 ya in the Middle East), goat (~ 10,500 ya in the Middle East), cattle (~ 10,300 ya in the Middle East), and horse (~ 5500 ya in central Asia) were domesticated to feed the farmer human^3^. It can be mentioned that Old World camels including dromedaries (1100–1800 BCE in Arabian Peninsula)^4^ and Bactrian camels (4450 ya in Central Asia)^5^ are the last domesticated animals among large mammals.

After domestication, artificial selection by humans along with climate-mediated selection were the most important factors for shaping the genetic and phenotypic structure of animals across different regions of the world. These factors have left their footprints on the specific region of the organism genome as the signature of selection. A decline in genetic variation has occurred in these regions followed by fixation of the selectively favorable alleles in the population after several generations^6^. Today, high-throughput techniques, availability of genomic data and advances in computational methods have made it possible to investigate the genomes of different organisms from various aspects.

Iran is one of the few countries that is home to both dromedary and Bactrian camels. From the past to the present, Bactrian camels have played a very important role in the lives of nomads inhabiting northwestern Iran (especially Ardabil province), as this area is one of the possible places for the domestication of Bactrian camels^5^. On the other hand, dromedary camels are spread in the central deserts of Iran and supply protein to many people in these areas. The devastating effects of climate change on the agricultural and livestock industry^7^ have necessitated the need for extensive studies on camels as one of the most resistant animals to harsh environmental conditions. It is a species that in the not-too-distant future could become a main source of food for large parts of Africa and Asia by becoming a farm animal.

In recent years, although many studies have been done to identify the selection signs in different species of domestic animals, including cattle^8,9^, sheep^10,11^, goat^12,13^ and horse^14,15^, but the studies of Bahbahani et al.^16^ on Sudanese dromedary camels and Liu et al.^17^ on Chinese Bactrian camels are among the few cases in this field for camels. In the present study, we used whole genome sequencing (WGS) data to identify the selection footprints on the genome of Iranian dromedary (ID) and Iranian Bactrian (IB) camels as a step to better understand these valuable species. Here, WGS data of dromedary camels from Arabian Peninsula (AP) and Bactrian camels from China-Mongolia (CM) were also used.

## Results

### Genomic data and SNP calling

Whole genome resequencing analysis was applied on 14 dromedary and 20 Bactrian camels to identify the signature of selection for ID and IB camels. A total of 2.23 and 2.67 billion cleaned reads were generated for dromedary and Bactrian camels, respectively. After removing duplicated reads, the mean depth was 15.64X and 14.27X for dromedary and Bactrian camels, respectively. We detected a total of 3,606,252 autosomal SNPs for dromedary and 6,992,165 autosomal SNPs for Bactrian camels (Table 1).

**Table 1:** Proportion of annotated SNPs in different genomic regions for dromedary and Bactrian camels.

Genomic region	Dromedary	Bactrian
Intron	1,558,384	3,071,665
Intergenic	1,438,870	2,782,614
Up/Downstream	407,780	753,105
Intragenic	94,139	189,288
UTR	47,158	89,849
EXON (Synonymous)	23,949	44,148
EXON (Non-Synonymous)	18,696	30,913
EXON (Start/Stop altering)	292	534
Spicing-Site	3879	6890
Others	13,105	23,159
Total	3,606,252	6,992,165

### Population genetic structure

**Figure 1:**
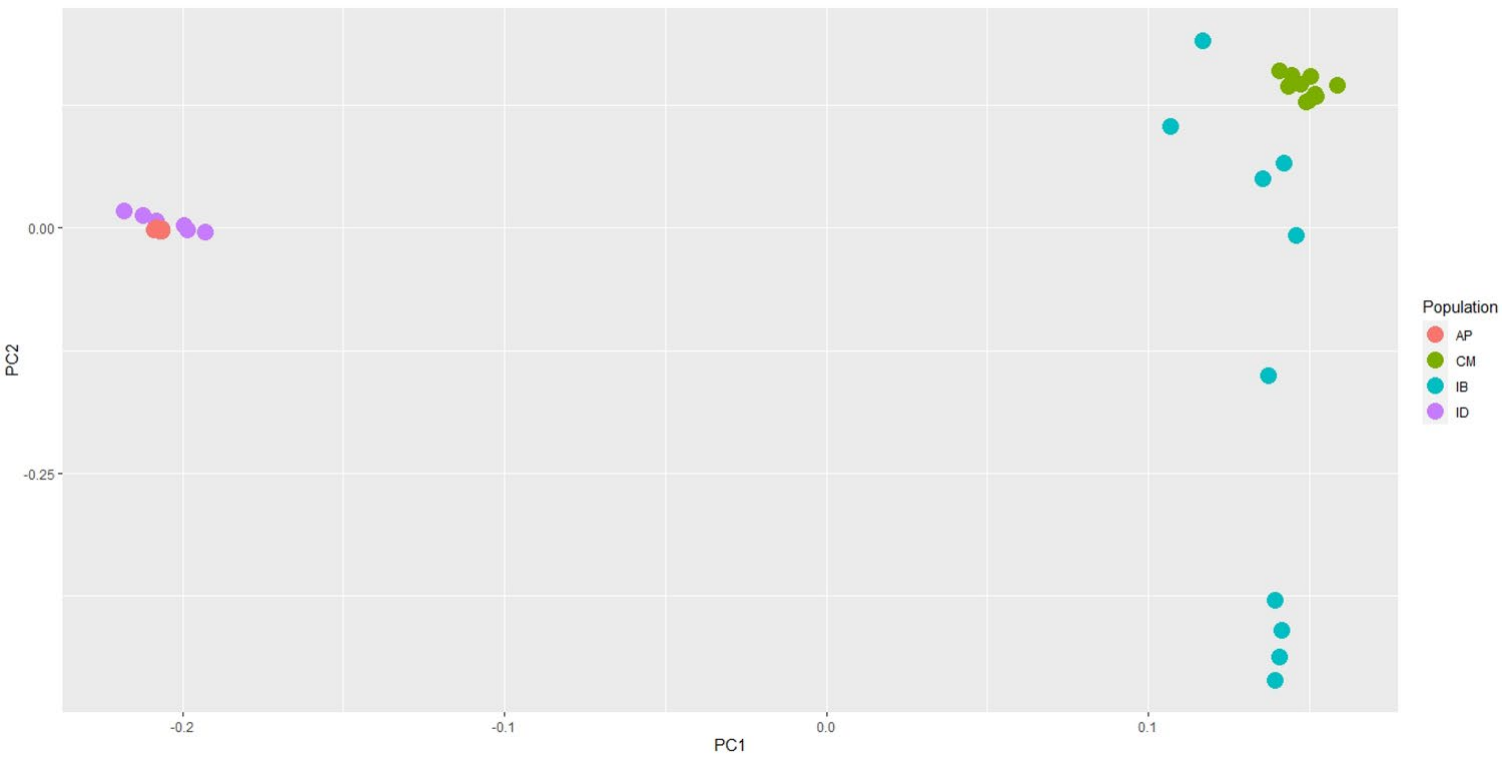
PCA plot visualized by R package ggplot2 (https://cran.r-project.org/web/packages/ggplot2); showing the two first PCs on all used dromedary and Bactrian camels in this study.

We used LD-pruned SNPs to obtain a general view of the genetic relationship between dromedary and Bactrian camels. As displayed in Fig. 1, PC1 separated two species and the PC2 reflects the genetic diversity among IB camels. Separation of IB from CM Bactrian camels is more clearly visible than ID from AP dromedary camels. In addition, PCA results for each species were presented in Supplementary Figs. S1 (dromedary camels) and S2 (Bactrian camels) separately.

### Genome-wide signature of selection

**Figure 2:**
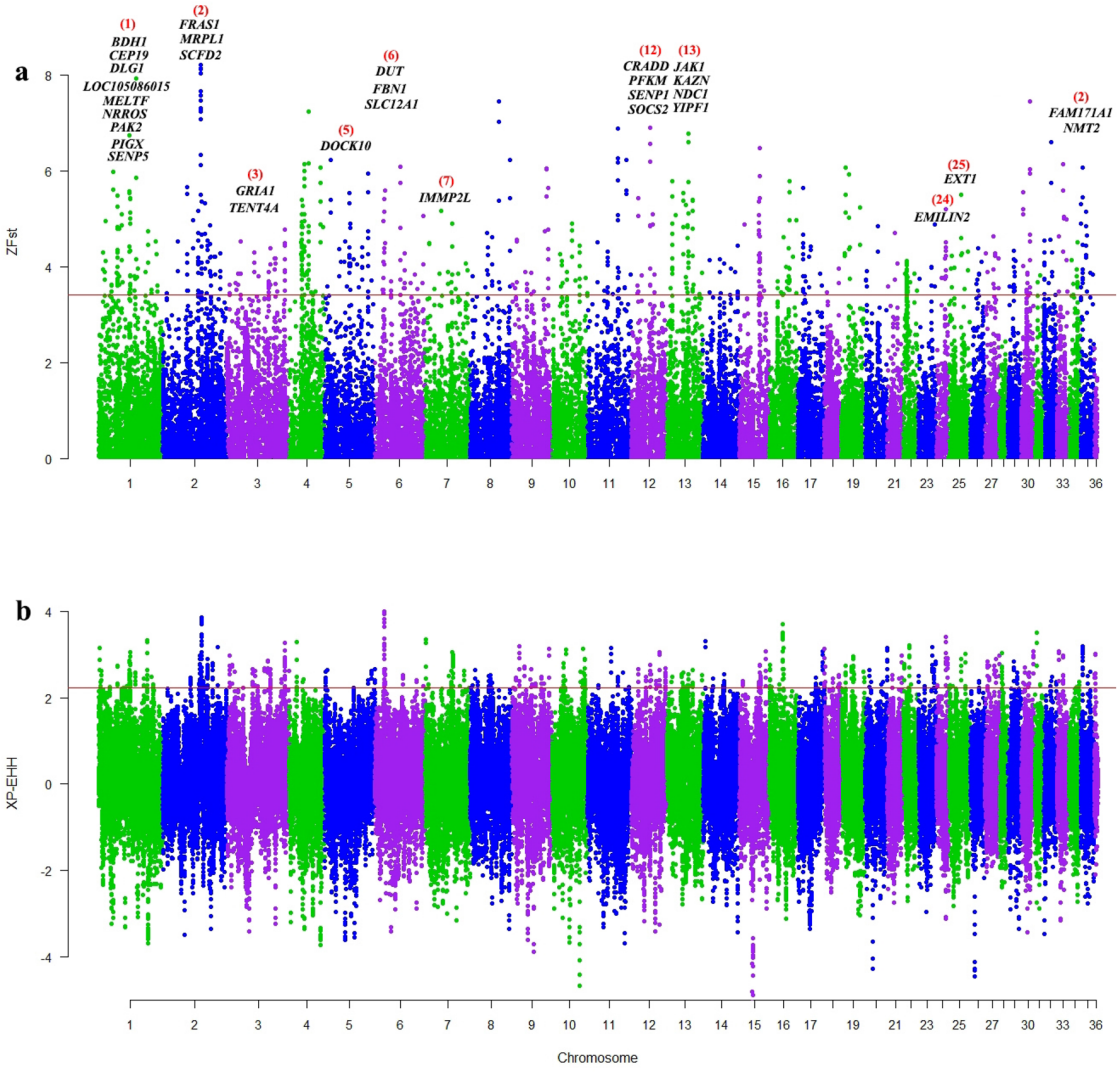
Manhattan plot visualized by R package qqman (https://cran.r-project.org/web/packages/qqman); showing the genome-wide distribution of ZFst (**a**) and XP-EHH (**b**) between ID and AP camels. The red line denotes a threshold of ZFst > 3.41 and XP-EHH > 2.24. The gene symbols inserted in the figure were identified as positively selected genes using both methods. The red numbers in parentheses represent the chromosome number.

**Figure 3:**
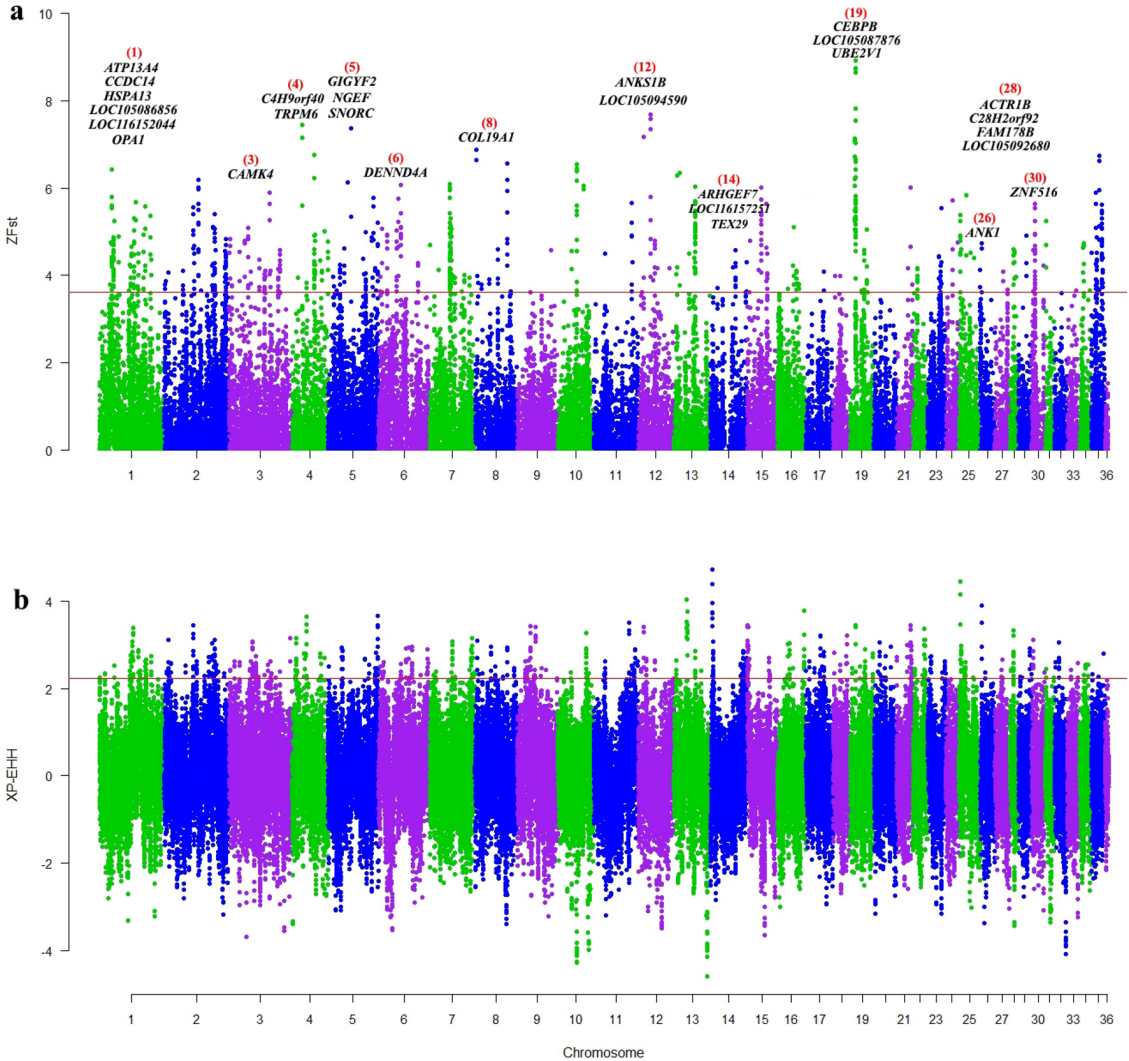
Manhattan plot visualized by R package qqman (https://cran.r-project.org/web/packages/qqman); showing the genome-wide distribution of ZFst (**a**) and XP-EHH (**b**) between IB and CM camels. The red line denotes a threshold of ZFst > 3.61 and XP-EHH > 2.23. The gene symbols inserted in the figure were identified as positively selected genes using both methods. The red numbers in parentheses represent the chromosome number.

Here we used two comparative sets including ID vs AP dromedary camels and IB vs MC Bactrian camels to identify the signature of selection for ID and IB camels, respectively. For both sets, genomic windows showing extremely high ZFst value and XP-EHH scores (top 1% percentile of distribution as threshold) were considered to be potential candidate regions under selection. Based on the results, we found 1525 (765 for ZFst and 760 for XP-EHH) and 1540 (769 for ZFst and 771 for XP-EHH) genomic windows with values higher than the 99th percentile of ZFst (ZFst > 3.41 for ID and ZFst > 3.61 for IB camels) and XPEHH distribution (XP-EHH > 2.24 for ID and XP-EHH > 2.23 for IB camels) as selective sweeps in ID (Fig. 2) and IB camels (Fig. 3), respectively.

Also, to identify selection signatures in AP and CM camels, Fst methods were applied on AP vs ID and CM vs IB sets. Then, genomic windows with scores higher than 99th percentile in ZFst distribution (AP vs ID and CM vs IB) and lower than first percentile in XP-EHH distribution (ID vs AP and IB vs CM) were defined as selection signatures for AP and CM camels. Based on the defined thresholds (ZFst > 3.41, XP-EHH < −2.19 for AP camels and ZFst > 3.61, XP-EHH < −2.17 for CM camels), we found 1534 (765 for ZFst and 769 for XP-EHH) genomic windows for AP (Supplementary Fig. S3) and 1549 (769 for ZFst and 780 for XP-EHH) genomic windows for CM (Supplementary Fig. S4) camels.

### Genes and GO analysis

**Figure 4:**
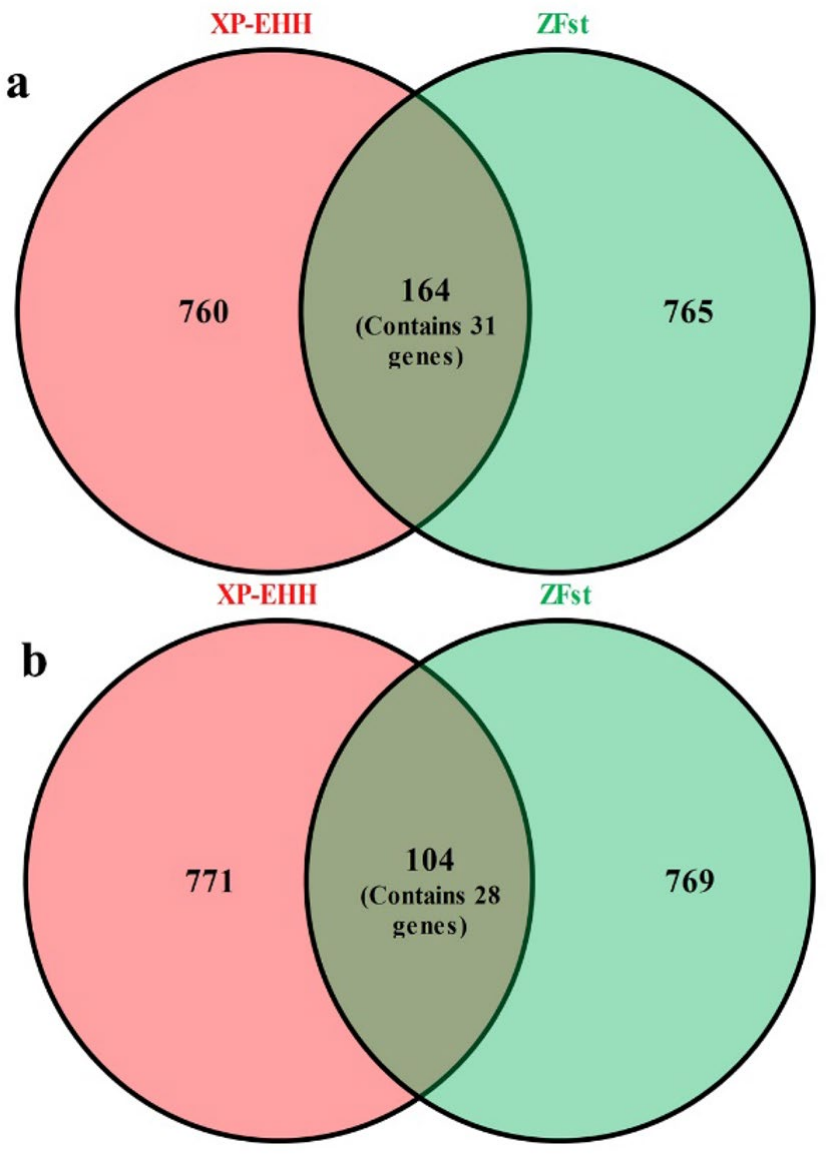
Venn diagram showing the overlapped genomic windows between the top 1% percentile of ZFst and XP-EHH distributions in ID (**a**) and IB (**b**) camels.

In total, we identified 649 (containing 450 protein-coding genes) genes from Fst and 521 (containing 369 protein-coding genes) genes from XP-EHH as candidate positively selected genes for ID camels. Also, 480 (containing 329 protein-coding genes) and 527 (containing 379 protein-coding genes) candidate genes were found for IB camels from Fst and XP-EHH, respectively. Furthermore, 164 (containing 31 protein-coding genes) and 104 (containing 28 protein-coding genes) overlapped genomic regions between the Fst and XP-EHH approaches were identified for ID and IB, respectively (Fig. 4). The genes located within those regions were considered as potentially under positive selection in Iranian camels. We performed GO analysis for combined unique protein-coding genes identified through both ZFst and XP-EHH statistics for ID vs AP (787) and IB vs MC (677) camels as well as for common genes identified by both methods for ID (31) (Supplementary Table S1) and IB (28) (Supplementary Table S2) camels. Based on the results, 18 (3 BP and 15 CC) (Supplementary Table S3) and 29 (26 BP and 3 CC) (Supplementary Table S4) GO terms were significantly enriched for combined unique genes for ID and IB camels, respectively. We also performed GO analysis on the genes shared between two used methods for ID and IB; 8 (7 BP and 1 CC) (Supplementary Table S5) and 21 (18 MF and 3 CC) (Supplementary Table S6) GO terms were found to be significantly enriched for ID and IB camels, respectively. Note that no significantly enriched pathways were found for any genes sets in the present study. Additionally, we found 34 (Supplementary Table S7) and 17 (Supplementary Table S8) genes as positively selected genes for AP and CM camels, respectively. The results of the GO analysis on these genes are presented in Supplementary Table S9 for AP and Supplementary Table S10 for CM.

## Discussion

As the most intelligent as well as the most ambitious species on earth, humans have tamed a number of animals, dominated them, and made significant changes in their phenotypes and genome architecture. Factors such as geography, climate, culture and even religion influence how humans interact with domestic animals. Thus, various domesticated animal breeds and ecotypes have been created by humans in different parts of the world for specific purposes. Now, the development of technologies such as WGS and high-density SNP arrays along with advances in the field of bioinformatics have enabled to identify footprints of positive selection in the genome of different species^18^.

Based on the PCA result, we observed high genetic diversity for IB camels compared with other populations in the PCA plot. This result was consistent with the findings based on mitochondrial sequence analysis which revealed that despite the small number of IB camels, they showed higher genetic diversity^19^. Also, based on microsatellite and WGS data, high heterozygosity was calculated for IB camels^5,20,21^.

Here, we used two inter-population approaches for detecting signatures of selection. Fst is one of the commonly used methods for identifying selective sweeps in domestic animals^18^ and considered as one of the effective approaches to detecting positively selected genes for populations without phenotypic data^22^. Indeed, by focusing on identifying differentiated parts of the genome between population, this method can detect recent signatures of selection^10^. According to Sabeti et al.^23^, XP-EHH identifies alleles that have been fixed in one population (target population) while being polymorphic in another (reference population) as selective sweeps. Generally, the XP-EHH scores range from negative to positive values, so a positive value suggests selection in the target population, while a negative value shows selection in the reference population.

Generally, the most of significantly enriched GO terms for IB camels were associated with development such as anatomical structure development (GO:0048856), lung alveolus development (GO:0048286) and central nervous system development (GO:0007417), along with immune system such as leukocyte activation (GO:0002366), natural killer cell cytokine production (GO:0002370), and lymphocyte activation (GO:0002285), as well as behavior such as adult behavior (GO:0030534), and adult locomotory behavior (GO:0008344). The only habitat of IB camels in Iran is in the area around Sabalan Mountain (4811 in elevation) located in Ardabil province. The nomads of this region (Shahsevan) as Bactrian camel owners, migrate to higher altitudes of the mountain in the warm season to provide suitable pasture for their sheep herds. In this nomadic lifestyle, most of the lifespan of IB camels is spent at high altitudes (up to 2500 m above sea level), which has probably led to the positive selection of genes involved in lung alveolus development for optimal use of oxygen. Of 26 GO terms in BP categories, six terms were associated with immune system in IB camels. In accordance with our findings for IB camels, several studies on different species^10,24,25^ have shown that immune-related genes have been among the targets of natural and artificial selection. Exposure to diverse pathogens in different regions may be the reason for the selection of different immune-related genes^26^.

Among the 28 genes located in the overlapped genomic windows between top 1% percentile of ZFst and XP-EHH scores, as the signature of selection for IB camels, the product of the ZNF516 gene as a cold-inducible factor activates UCP1 transcription to promote browning of white fat and the development of brown fat in mice^27^. Since, brown adipose tissue acts as a thermal regulator and heat generator for the body upon cold exposure^28,29^, the positive selection of gene involved in the development of this tissue can be vital for Bactrian camels that have adapted to cold climates. The CAMK4 gene is known as a domestication-related gene in sheep^30^ and has been shown to play an important role in the reproduction of mice^31^, sheep^32^, and chicken^33,34^. This gene is also associated with the formation of long-term memory^35,36^. One of the interesting genes was the ANK1 which was found to have a link between polymorphism in its promoter region and the meat quality of porcine^37^ and cattle^38,39^. Additionally, the HSPA13 gene influences meat quality by affecting the water holding capacity of meat^40^. The results of some studies^41,42^ have indicated an association between the ANKS1B gene and growth plus body size in sheep. COL19A1 and NGEF play roles in muscle differentiation^43^ and fat deposition^44^, respectively. Evidence suggests that the TRPM6 gene is involved in magnesium homeostasis and absorption in the intestine and kidneys^45^. The results of a study showed that a magnesium-deficient diet causes hypomagnesemia and enhances the expression of the TRPM6 gene in mice kidney and intestine^46^. One of the possible reasons for the selection of this gene in IB camels may be related to the composition of soil and vegetation in the habitat of camels; however, to confirm this claim we need accurate information about these parameters. Previous studies have found that the OPA1 gene, as one of the candidate genes under selection for IB camels, is involved in the adaptation of snow and Tibetan sheep to survive at high altitudes with low oxygen and temperature^47,48^, that is somewhat similar to the habitat of IB camels. Also, Tang et al.^49^ reported that OPA1 potentially enhances the ability of Tibetan chicken to survive under the hypoxic condition of the Qinghai-Tibet Plateau.

Among the 31 genes located in the overlapped genomic windows between top 1% percentile of ZFst and XP-EHH scores, as the signature of selection for ID camels, the BDH1 plays an important role in energy metabolism through synthesis and degradation of ketone bodies. During starvation, the ketone bodies are considered one of the most important sources of energy for the body^50^. The protein encoded by this gene as one of the ketone body metabolic enzymes, can be a key factor in the efficiency of camels for the optimal use of ketones. Another candidate under selection genes in ID camels was CEP19. Yang et al.^51^ found that this gene as a protein synthesis related gene was one of the positively selected genes in fox and probably plays a role in adaptation to strong seasonal fluctuation and energy requirements. Inactivation of this gene could lead to morbid obesity in humans and mice^52^. The CRADD is associated with muscle compactness and is well-known as a meat quality related gene^53,54^. DLG1, IMMP2L and, FRAS1 were three reproduction-related genes that we found in this study as the putative signature of selection in ID camels. Hernández-Montiel et al.^55^ reported that the DLG1 gene may be associated with the ovary development process and litter size in ewes. The IMMP2L gene plays a role in fertility in mice^56,57^. Also, it has been found that the FRAS1 gene is one of the candidate genes for cattle^58^ and pig^59^ fertility. EMILIN2 (belonging to elastin protein family) and FBN1 (belong to fibrillin protein family) are involved in fibrogenesis in the yak lung which along with several other genes can improve the efficiency of oxygen utilization in yaks at high altitude^60^. It has been found that GRIA1 is one of the domestication-related genes in the dog^61^ and cat^62^. The protein encoded by this gene, known as neurotransmitter receptor, plays an important role in the long-term memory formation^63^. We hypothesized that the identification of this gene as one of the candidate genes under selection in ID camels is probably due to their rearing system in Iran. ID camels in the central region of Iran are left in the desert every spring to graze^64^, and a memory-related gene (GRIA1) can be linked to finding pre-identified food sources as well as returning home. The SLC12A1 gene is one of the important kidney-related genes that we found as one of the positively selected genes in ID camels. Mara et al.^65^ reported that this gene is a key determinant of physiological water conservation in desert rodents, due to its role in the reabsorption of sodium and subsequent water reabsorption in the kidney. Several genes including SOCS2, JAK1, NRROS, and SENP1 were immune-related genes^66–69^. However, evidence suggests that the SOCS2 gene is potentially associated with milk production traits^70^, growth, body weight^71^, as well as efficient energy metabolism^72^.

The GO analysis to functionally categorize the positively selected genes for CM camels led to the identification of three GO terms related to embryo development including embryo implantation (GO:0007566), female pregnancy (GO:0007565) and positive regulation of endodermal cell differentiation (GO:1903226). The obtained results showed differences in the selection pattern in IB and CM camels. This difference in selection pattern can be related to various factors such as geography, climate and also the culture of camel breeders in Iran and China-Mongolia. Investigation of selection signatures in AP camels revealed that three olfactory-related genes (LOC105087657, LOC105105401 and LOC105105413) are on the list of identified genes. Presumably, the role of olfactory receptors in locating food and mate, avoiding threats (e.g. predators and toxins) and feeding behaviors (e.g. food choice)^73^ has made these genes a candidate to help AP camels adapt to harsh desert environments.

## Conclusion

In the present study, we used WGS data of dromedary (from Iran and AP) and Bactrian camels (from Iran and MC) to detect the genome-wide signature of selection in the ID and IB camels. Using two methods, we identified genomic regions under positive selection that harbor some considerable genes in ID and IB camels. Despite the interesting results, the small sample size can be considered as a limitation in the present study. Therefore, further studies with a higher number of samples along with other levels of omics data (e.g. RNA-Seq data) can help to better understand the genetic basis of production-related phenotypes of camels. It can be mentioned that camels have not been subjected to intense artificial selection, unlike domestic animals such as cattle, sheep, horse, chicken and pig. Therefore, knowledge about genomic features of camels can be an important step in designing and implementing effective breeding programs for camels in the future. Indeed, understanding the mechanism of selection, genetic background of adaptation processes to local environment as well as identification of candidate genes of interest to breeding programs can help us to draw a roadmap for camel breeding in the near future.

## Materials and methods

### Samples and whole genome resequencing

WGS data of 34 Old World camels including 14 dromedaries and 20 Bactrian camels were used in the present study. Of these samples, 6 and 10 samples belonged to ID and IB camels, respectively. ID samples were from Semnan (latitude: 35.429288 and longitude: 55.007100), Yazd (latitude: 31.610649 and longitude: 55.447436) and Ardabil (latitude: 38.249364 and longitude: 48.297162) provinces, while samples of IB were from Ardabil province as the only habitat of Bactrian camels in Iran. Additionally, to use as reference population against ID and IB camels to identify signature of selection, WGS data of 8 dromedary camels belonging to AP and 10 Bactrian camels belonging to CM were obtained from Sequence Read Archive at the NCBI (Supplementary Table S11).

For sequencing, after shearing of genomic DNA, fragments with 300-500 bp were generated after which these fragments were repaired, ‘A’-tailed, and ligated to Illumina sequencing adapters. DNA fragments with 370-470 bp were selected on 2% agarose gels and after amplification using PCR, were used for sequencing. Finally, one lane of 150 bp paired-end sequencing was performed by Illumina HiSeq 2500 system.

### Raw reads filtration

Before performing any analysis, the quality of the sequenced reads was assessed by FastQC v.0.11.9 (https://www.bioinformatics.babraham.ac.uk/projects/fastqc/). We used Trimmomatic v.0.39^74^ to apply quality filtration on raw reads using the sliding windows (5:20) approach. The employed trimming options were not the same for all samples as HEADCROP and ILLUMINACLIP were applied to some samples for removing base content noises and adapter contamination, respectively. Finally, reads with less than 40 bp were excluded from downstream analysis.

### Alignment and variant calling

Reads that passed the quality filtration step, were mapped to the dromedary camel reference genome (GCF_000803125.2) using BWA v.0.7.17^75^ with the “mem” algorithm. The outputted SAM file was converted to BAM format using Samtools where sorting of BAM file in coordinate order was done by Picard “SortSam” (http://broadinstitute.github.io/picard). Mark duplication was performed using Picard to remove duplicated reads during sample preparation and sequencing steps. Two approaches of Genome Analysis Toolkit (GATK) v.3.7^76^ including “RealignerTargetCreator” and “IndelRealigner” were performed for local realignment around INDELs.

In the present study, to identify single nucleotide polymorphisms (SNPs), GATK “HaplotypeCaller” algorithm with “-ERC GVCF” mode was used to create “gVCF” for each sample separately. Then, we used “GenotypeGVCFs” approach of GATK to jointly genotype all gVCFs for creating a VCF file containing raw called variants (SNPs and INDELs) for all samples in each group (dromedary and Bactrian camels). Called SNPs were extracted from the VCF file by GATK “SelectVariants” for further analysis. The identified SNPs were filtered using BCFtools, VCFtools^77^, and GATK with the following criteria: SNPs with QD < 2, FS > 60, MQ < 40, MQRankSum < −12.5, ReadPosRankSum  < −8 and SOR > 3 were removed. We also filtered those SNPs with a cluster windows size of 10 bp and a cluster size of 3 SNPs. Finally, SNPs with only two alleles, sites with minor allele frequency greater than or equal to 0.05 and loci with genotypes called in at least 80% of individuals were retained for downstream analysis. We then excluded chromosome X and unplaced contigs from the final VCF file. Annotation of final SNPs was carried out using SnpEff v.5.0e^78^.

### Population genetic analysis

The genetic relationship between dromedary and Bactrian camels was investigated using principal component analysis (PCA) by PLINK1.9^79^. Before performing PCA, we pruned identified SNPs using the “indep-pairwise 50 10 0.2” option in PLINK1.9 to minimize the effects of SNPs contributed by regions of extensive strong linkage disequilibrium (LD). This would remove one of the pairs of SNPs with a square correlation greater than 0.2 in widows with 50 and a step size of 10 SNPs.

### Detection of selection signatures

To identify regions under positive selection in the genome of ID and IB camels, we used two methods including population differentiation index (Fst) and cross-population extended haplotype homozygosity (XP-EHH) with 50 kb sliding window and 25 kb step size. The window size and step size were selected for this study based on previous studies on the genomes of dromedary^16^ and Bactrian^17^ camels. The Fst in each window and XP-EHH score for each SNP were estimated using VCFtools and Selscan v.2.0^80^, respectively. In the present study, Beagle v.5.2^81^ was performed with argument burnin = 5 and iterations = 20 for haplotype phasing. For dromedaries, ID camels constituted the target population while dromedaries from AP formed the reference population. In the second comparative set, IB camels were regarded as the target population compared to CM Bactrian camels as the reference population.

Normalization of each XP-EHH score across all chromosomes was performed using norm program distributed along with Selscan. We transformed Fst values to ZFst: ZFst = (Fst – μFst)/σFst, where μFst and σFst are the mean and standard deviation of Fst values in all windows. Note that windows containing ≤ 5 SNPs were removed to reduce the probability of identifying false positive selected regions. We estimated the average normalized XP-EHH score in each window for both comparative sets (ID vs AP and IB vs CM). The overlapped genomic windows in the top 1% percentile of ZFst values and normalized XP-EHH scores were considered as the signature of selection in the ID and IB genomes. For a comparative view, a similar process was performed for the other two sets, including AP vs ID and CM vs IB.

### Annotation and gene ontology

The putative selective sweeps identified by Fst and XP-EHH methods were annotated using GTF (gene transfer format) file related to dromedary camel genome and BEDtools v.2.27.1^82^. In each comparative set, for gene ontology (GO) and Kyoto Encyclopedia of Genes and Genomes (KEGG) pathway analysis, we submitted the combined list of the identified genes by Fst and XP-EHH approaches, as well as a list of genes shared between the two mentioned approaches to “g:Profiler”. GO analysis allows us to better understand and express the biological function of genes and classifies them into three categories including biological process (BP), molecular function (MF), and cellular component (CC). The calculated p-values were corrected using the Benjamini–Hochberg FDR (false positive rate) and enriched terms were considered statistically significant at p-adjusted ≤ 0.05.

## Supplementary Information

Supplementary Information 1.

Supplementary Information 2.

## Data Availability

Three new whole genome sequence data related to Iranian dromedary (one sample) and Bactrian (two samples) camels analyzed in this study are available in the Sequence Read Archive (SRA) with the BioProject accession number PRJNA801784 (including SRR17817908, SRR17817909 and SRR17817910).
