# Supplementary material for: Identification of selection signatures in Iranian dromedary and Bactrian camels using whole genome sequencing data
Source: Sci Rep. 2022 Jun 10;12:9653. doi: 10.1038/s41598-022-14376-7 (PMC9187634; doi:10.1038/s41598-022-14376-7)
Supplement: Supplementary file 1 — Supplementary Information 1. [file 41598_2022_14376_MOESM1_ESM.pdf]

Identification of selection signatures in Iranian dromedary and Bactrian camels using whole genome sequencing data

Supplementary material

Reza Khalkhali-Evrigh[1], Nemat Hedayat[1*], Liang Ming[2] & Jirimutu[2]

[1]Department of Animal Science, Faculty of Agriculture and Natural Recourses, University of Mohaghegh Ardabili, Ardabil, Iran
[2] College of Food Science and Engineering, Inner Mongolia Agricultural University, Huhhot, China

[*]Correspondence to Nemat Hedayat (nhedayat@uma.ac.ir)

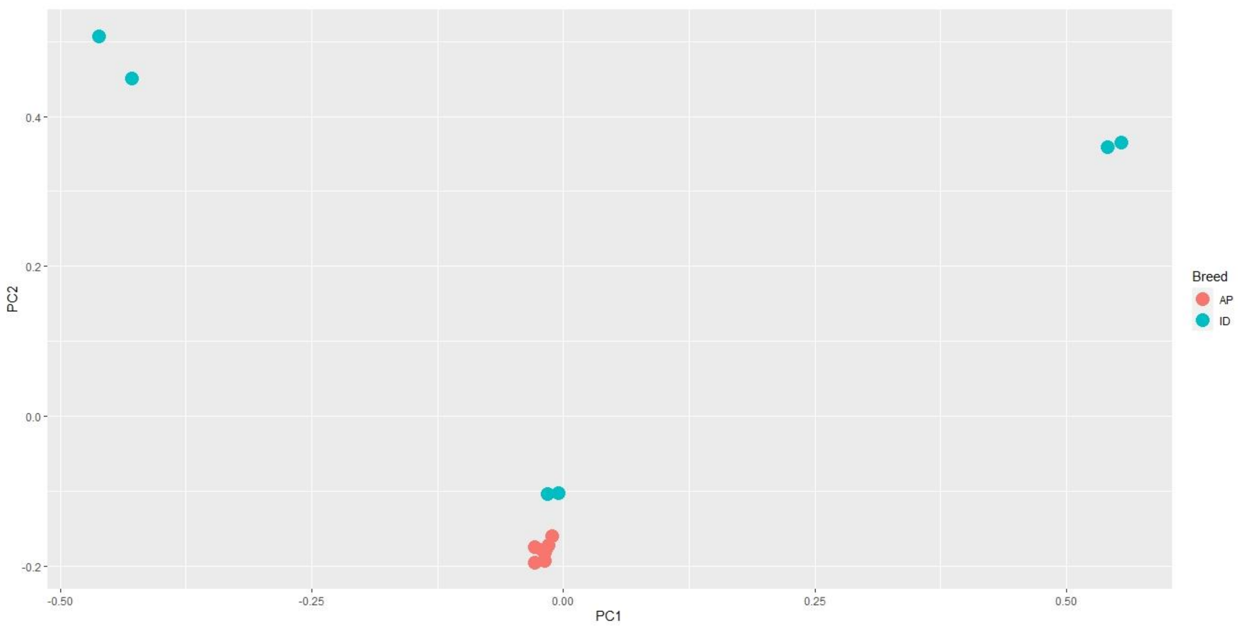

Supplementary Figure S1: PCA plot visualized by R package ggplot2 (https://cran.r-project.org/web/packages/ggplot2); showing the two first PCs on dromedary samples

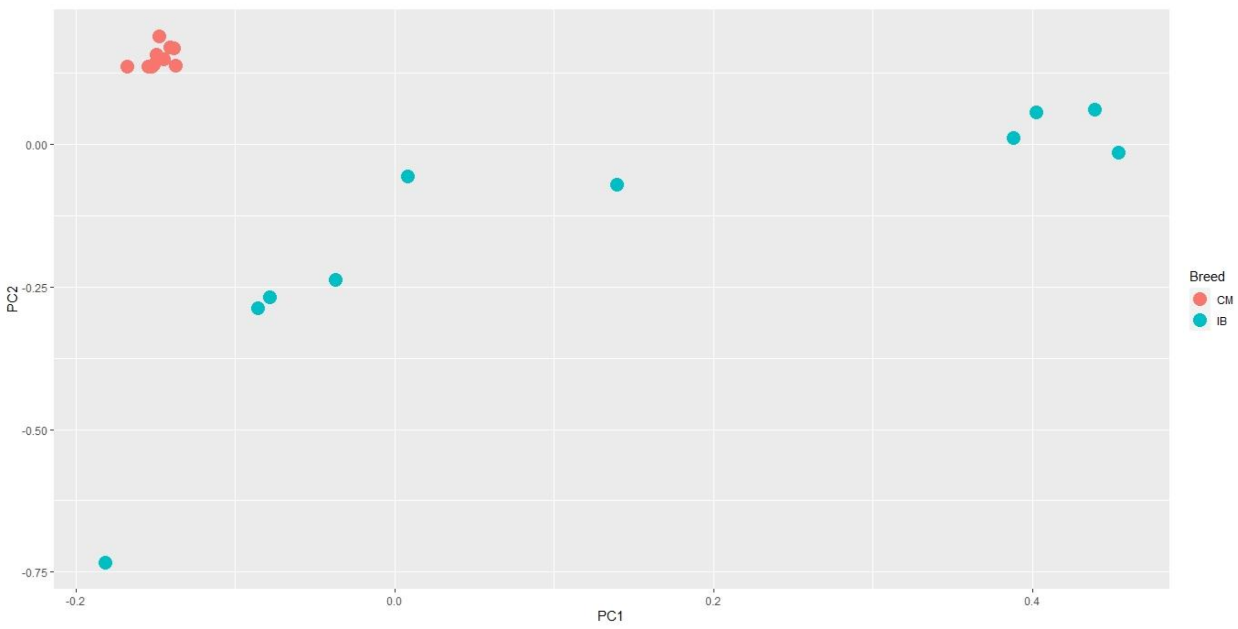

Supplementary Figure S2: PCA plot visualized by R package ggplot2 (https://cran.r-project.org/web/packages/ggplot2); showing the two first PCs on Bactrian samples

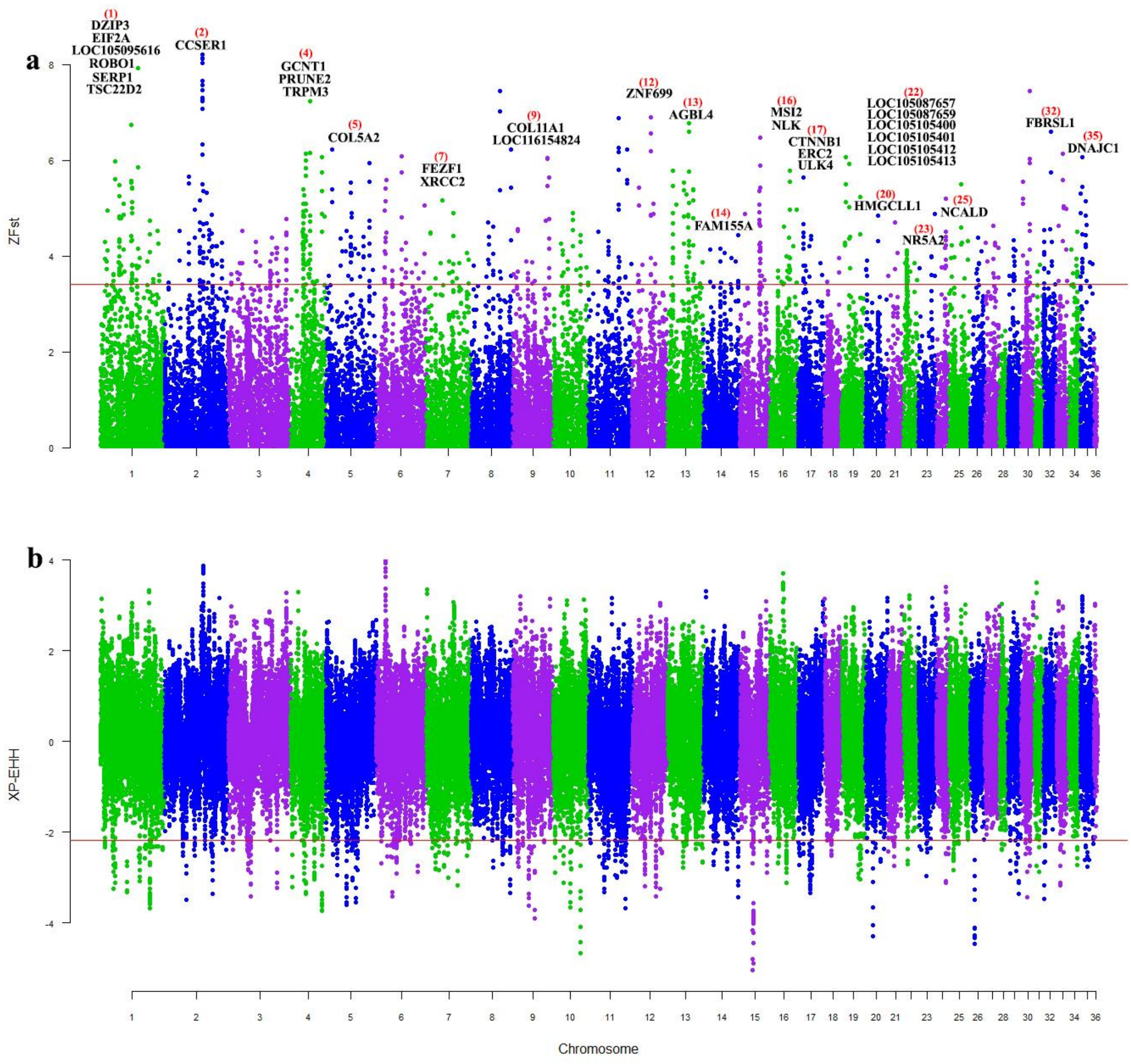

Supplementary Figure S3. Manhattan plot visualized by R package qqman (https://cran.r-project.org/web/packages/qqman); showing the genome-wide distribution of ZFst (a) and XP-EHH (b) between AP and ID camels. The red line denotes a threshold of ZFst > 3.41 and XP-EHH < -2.19. The gene symbols inserted in the figure were identified as positively selected genes using both methods. The red numbers in parentheses represent the chromosome number.

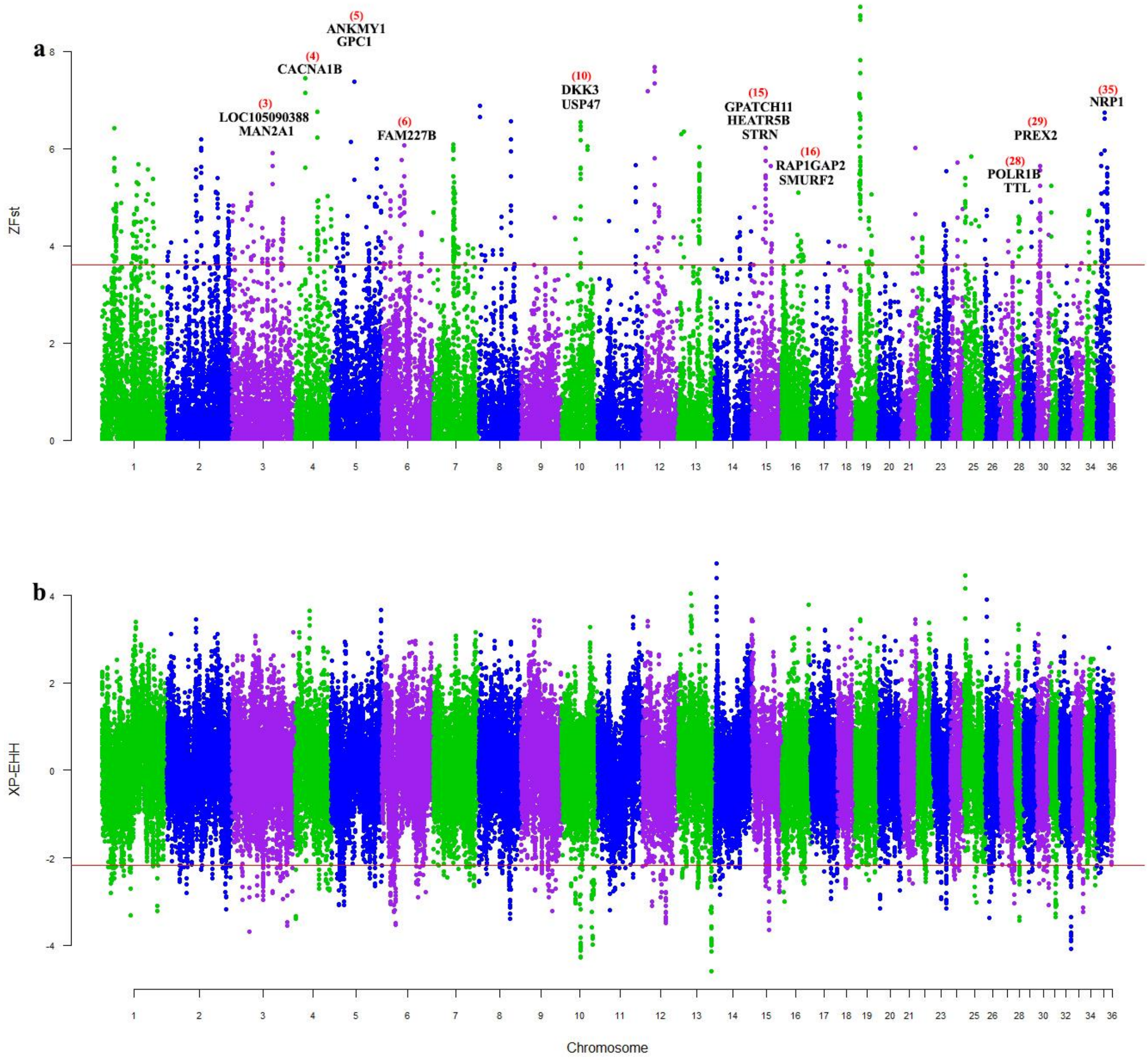

Supplementary Figure S4. Manhattan plot visualized by R package qqman (https://cran.r-project.org/web/packages/qqman); showing the genome-wide distribution of ZFst (a) and XP-EHH (b) between CM and IB camels. The red line denotes a threshold of ZFst > 3.61 and XP-EHH < -2.17. The gene symbols inserted in the figure were identified as positively selected genes using both methods. The red numbers in parentheses represent the chromosome number.
